# Life-threatening dermatoses: Stevens-Johnson Syndrome and Toxic Epidermal Necrolysis. Impact on the Spanish public health system (2010-2015)

**DOI:** 10.1371/journal.pone.0198582

**Published:** 2018-06-18

**Authors:** Virginia Velasco-Tirado, Montserrat Alonso-Sardón, Adriana Cosano-Quero, Ángela Romero-Alegría, Leire Sánchez-los Arcos, Amparo López-Bernus, Javier Pardo-Lledías, Moncef Belhassen-García

**Affiliations:** 1 Servicio de Dermatología. Complejo Asistencial Universitario de Salamanca, Salamanca, Spain; 2 Instituto de investigación Biomédica de Salamanca, Salamanca, Spain; 3 Centro de Investigación de Enfermedades Tropicales de la Universidad de Salamanca, Universidad de Salamanca, Salamanca, Spain; 4 Área de Medicina Preventiva y Salud Pública, Facultad de Medicina, Universidad de Salamanca, Salamanca, Spain; 5 Servicio de Medicina Interna, Complejo Asistencial Universitario de Salamanca, Salamanca, Spain; 6 Unidad de Enfermedades Infecciosas, Servicio de Medicina Interna. Complejo Asistencial Universitario de Salamanca, Salamanca, Spain; 7 Servicio de Medicina Interna. Hospital Marques de Valdecilla, Santander. Avenida Valdecilla. Santander, Spain; NYU Langone Medical Center, UNITED STATES

## Abstract

**Background:**

Stevens-Johnson Syndrome (SJS) and Toxic Epidermal Necrolysis (TEN) are serious mucocutaneous reactions. In Spain, the epidemiology and resulting expenses of these diseases are not well established.

**Methodology:**

Retrospective descriptive study using the Minimum Basic Data Set (CMBD in Spanish) in patients admitted to hospitals of the National Health System between 2010 and 2015 with a diagnosis of SJS and TEN (combination of ICD-9 codes 695.13, 695.14, and 695.15, along with length of hospital stay).

**Principal findings:**

A total of 1,468 patients were recorded, 773 were men (52.7%). The mean age (± SD) was 52.25 ± 26.15 years. The mean incidence rate for all diagnoses was 5.19 cases per million person-years (2.96 in SJS, 0.31 in SJS/TEN and 1.90 in TEN). 148 patients died (10.1%), 47 due to SJS (5.6%) and 90 (16.7%) due to TEN. The estimated total medical cost of SJS, SJS/TEN, and TEN in Spain was €11.576.456,18, and the average medical cost per patient was €7.885,86 ± €11.686,26, higher medical cost in TEN (€10352.46 ± €16319,93) than in SJS (€6340,05 ± €7078,85) (p<0.001).

**Conclusions:**

Older patients have a more severe clinical picture and higher mortality rates. The overall mortality of both diseases is approximately 10%, and clinical diagnosis and age were the variables with the greatest influence on mortality. This study describes a stable incidence and a similar prevalence to other European countries. Additionally, the data show a high cost due to hospitalizations. Finally, the CMBD could be a good system of epidemiological analysis for the study of infrequent diseases and hospital management of conditions such as SJS and TEN.

## Introduction

Stevens-Johnson Syndrome (SJS) and Toxic Epidermal Necrolysis (TEN) are rare mucocutaneous reactions that can be quite severe and are triggered mainly by medications. Infectious diseases are secondary triggers of these pathologies, especially in the pediatric population, with *Mycoplasma pneumoniae* infection the most prevalent[1]. The list of responsible drugs is very broad, with carbamazepine, phenobarbital, phenytoin and allopurinol being the most frequently implicated[2]. At present, SJS and TEN are considered variants of the same entity[3]; either of them can occur at any age, and in most cases, they are acute and self-limited conditions. Clinically, they are characterized by fever, general symptoms and detachment of the epidermis. The usual management is hospitalization, and depending on the degree of cutaneous involvement (skin detachment greater than 30% of the body surface area), the clinical situation or associated comorbidities, it may require entry into intensive care units. TEN and SJS have high rates of morbidity and mortality, and prognostic factors include age, extent of detachment, visceral involvement, neutropenia, uraemia and hyperglycaemia. The SAPS, SAPS 11 and the SCORTEN are the common prognostic systems that are validated and applied clinically [4]. Mortality rates vary: 30–35% in TEN, 10–15% in cases of SJS/TEN, and 5% in SJS[5]. Mortality is mainly due to complications secondary to SJS or TEN, such as sepsis, acute pulmonary oedema, pulmonary thromboembolism and digestive haemorrhage[6].

Epidemiological information on this group of diseases is very limited. The incidence of TEN and SJS described in some European countries such as France and Germany varies between 1–1.4 and 1–3 cases x 10^6^ inhabitants/year, respectively[7–12], while the incidence is somewhat lower in Asian countries[13]. It should be noted that SJS and TEN occur more frequently in women compared to men, with a ratio of men to women of 0.6[14], and the incidence of SJS and TEN is approximately 100 times higher in individuals with Human Immunodeficiency Virus (HIV) than in the general population[15]. Data regarding the economic impact of TEN and SJS are scarce, and in Spain, the clinical and epidemiological characteristics and the hospital expenditures incurred as a result of this group of diseases are unknown. The objective of this study is to evaluate the impact of SJS and TEN in the Spanish National Health System during the period from 2010–2015.

## Material and methods

### Study population and data source

This study was a retrospective longitudinal descriptive study of hospitalized patients diagnosed with SJS and TEN in public hospitals of the Spanish National Health System (NHS) between 2010 and 2015. Spain’s NHS is characterized by its publicly funded universal coverage and by having a broad portfolio of services that includes all technologies and health procedures with which scientific knowledge faces diseases and their consequences on human health, and it is composed of all the Health Services of the State Administration and the Health Services of the 17 Autonomous Communities that make up the Spanish State.

Data were obtained from the Minimum Basic Data Set (CMBD in Spanish). These data were provided by the Health Information Institute of the Ministry of Health and Equality. The CMBD is a mandatory and common administrative registry in all public hospitals in Spain, which contains a set of clinical, demographic and administrative variables that provide information about the users, the centres and units that attend to the patient and their care process. It contains 19 compulsory variables of which the most important are: age, sex, main diagnosis, secondary diagnoses, procedures, and circumstances regarding hospital discharge. As the “**Primary/main diagnosis**”, it refers to the condition that at the end of the hospitalization process is considered the cause responsible for the patient's admission to the hospital. “**Secondary diagnoses******” (up to 13) are the diagnoses that coexist with the main diagnosis at the time of admission or develop during it. **Circumstance to hospital discharge** refers to the patient leaves the hospital alive or dead, or is transferred to another hospital or voluntary discharge. **Re-entry** refers to the existence of an urgent-type income in a time less than or equal to 30 days following the date of discharge of the index episode, regardless of what the diagnosis was upon discharge. **Costs** are an estimate of the consumption of resources and costs, called *relative weights*, which are obtained from the information on the costs of hospital care, obtained by the analytical accounting systems.

### Selection of SJS, SJS/TEN, and TEN

We used the *International Classification of Disease*, *9*^*th*^ *edition*, *Clinical Modification* (ICD-9-CM) codes to identify SJS, SJS/TEN, and TEN: SJS, code 695.13;, code 695.14; and TEN, code 695.15). Furthermore, based on a previously validated algorithm, patients who had a *length of hospital stay* (LOS) shorter than 3 days were excluded to improve the positive predictive value of the diagnostic codes[16].

We **included** all patients admitted to public hospitals of the NHS between 2010 and 2015 with a primary and/or secondary diagnosis of SJS, SJS/TEN, and TEN according to the ICD-9-CM codes, along with a LOS shorter than 3 days. We **excluded** those patients with LOS for 2 or fewer days. Patients with missing data were also excluded from the study. Firstly, 1,765 patients with diagnosis of SJS, SJS/TEN, and TEN were selected during the study period. Of these, 297 patients had a LOS shorter than 3 days, and they were excluded. Finally, 1,468 patients were included in the study.

There is growing evidence that SJS/TEN in the pediatric population has different epidemiology and outcomes than adults, so we stratify the sample in pediatric (<16 years old) vs adult (≥16 years old).

### Incidence rates

Incidence rates were calculated by dividing the number of new SJS, SJS/TEN, and TEN cases observed in the defined time period 2010–2015 by the total number of disease-free periods in person-time during the observation period defined in the study (6 years) multiplied per 1,000,000 and expressed as 10^6^ *“cases per million person-years”*. Since it is not possible in this case to accurately measure disease-free periods, the total value of person-time at risk can be roughly and satisfactorily estimated; when the population size is stable, the average population size under study is multiplied for the duration of the observation period. The denominators were obtained from the annual population figures of the register published by National Statistical Institute (INE in Spanish) (http://www.ine.es/) Average estimated population of Spain, period 2010–2015: 47,129,783 inhabitants, pediatric population (<16 years old) 7,511,900, and adult population (≥16 years old) 39,617,883 inhabitants. 95% confidence interval (95% CI) for incidence rate has been calculated for a better interpretation and clinical application of the results.

### Statistical analysis

The statistical analysis includes a descriptive analysis of each variable. Quantitative results are expressed as the mean and standard deviation (SD). The qualitative results are expressed in absolute and relative (percentage) values. Subsequently, a bivariate analysis was performed to study the influence of the different clinical and epidemiological variables collected on the dependent variables. The strength of the association between the qualitative variables was measured using Pearson’s χ^2^ contrast statistic and the Odds Ratio (OR) estimate. For the comparison of means in independent groups, Student’s t test was used. For the comparison of means in more than two independent groups, ANOVA variance analysis was used. Finally, to jointly analyse the clinical and epidemiological variables that influence the mortality of SJS and TEN, we performed a multivariate logistic regression analysis, estimating parameter B, its standard error (SE), its statistical significance with the Wald test and the estimate of the OR (Exp (B)) with its 95% CI. The level of statistical significance was p < 0.05, the confidence intervals were 95%, and the statistical package used was SPSS 23.0.

### Ethics statement

This study involves the use of medical data from CMBD patients. These data are organized by the Ministry of Social Services of Health and Equality (MSSSI in Spanish). Researchers working in public and private institutions can request databases by completing a questionnaire available on the MSSSI website. In this questionnaire, a signed confidentiality commitment is required. All patient data provided by the CMBD are anonymised and unidentified by the MSSSI before they are provided to the applicants. According to this confidentiality commitment signed with the MSSSI, researchers cannot provide the data to other researchers, so other researchers must request the data directly from the MSSSI.

The study protocol was approved by the Clinical Research Ethics Committee of the Complejo Asistencial Universitario de Salamanca (Salamanca, Spain). The procedures described here were carried out in accordance with the ethical standards described in the Revised Declaration of Helsinki in 2013.

## Results

### Incidence rates

A total of 1,468 patients were included during the study period, 773 (52.7%) patients were male, and 695 (47.3%) were female. The incidence rate in Spain obtained in this study for these three diagnoses together is 5.19 cases per million py (95% CI, 4.92–5.45): 2.96 cases/million py (95% CI, 2.76–3.16) in SJS, 1.90 cases/million py (95% CI, 1.74–2.07) in TEN, and 0.31 cases/million py (95% CI, 0.24–0.38) in SJS/TEN. The incidence rate is slightly higher in men than in women, 5.55 (95% CI, 5.16–5.94) *vs* 4.83 (95% CI, 4.47–5.19) cases/million py.

**Fig 1:**
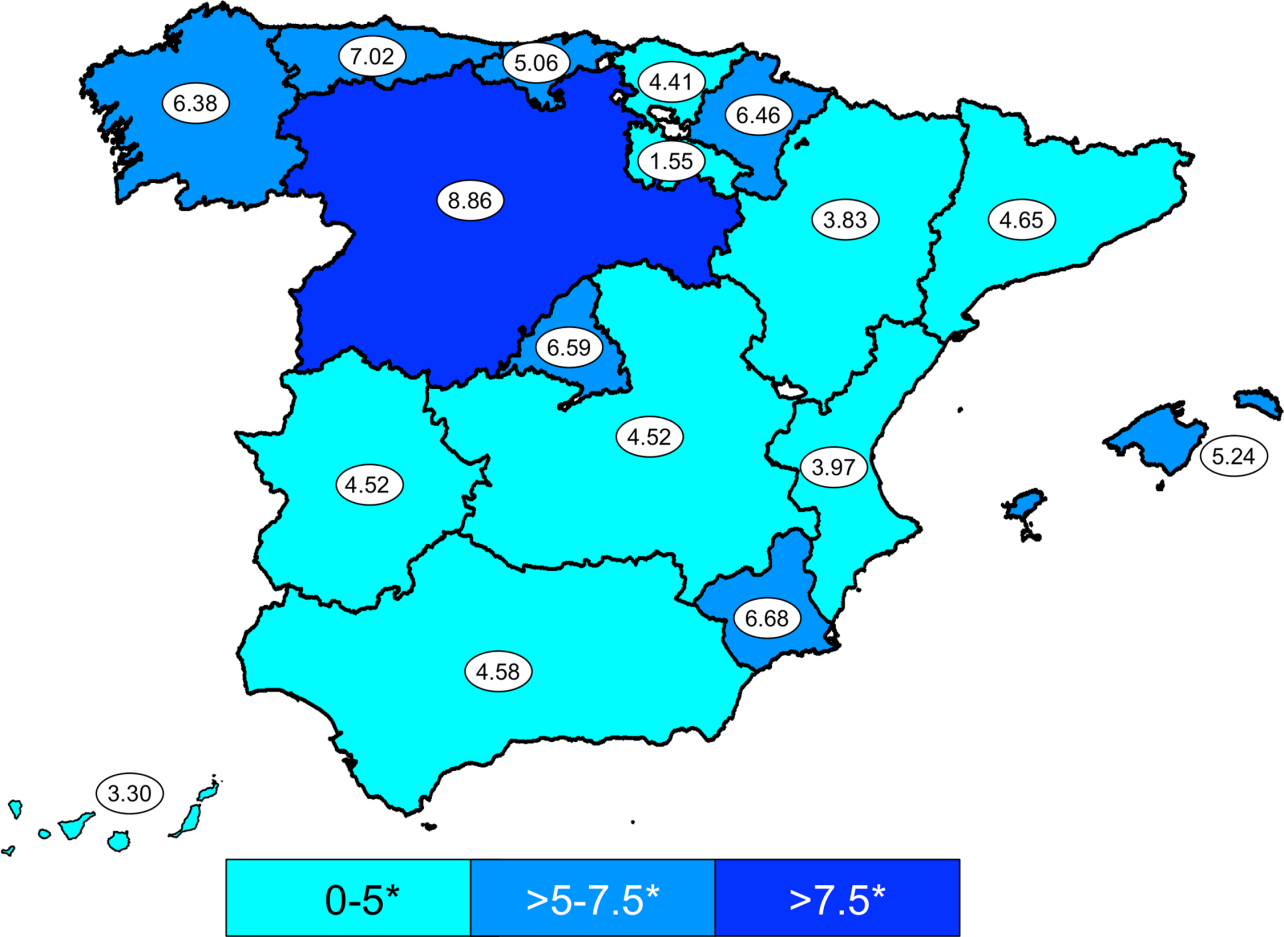
Incidence rates of SJS, SJS/TEN, and TEN in the Autonomous Communities of Spain.

Two hundred and seven patients (14.1%) were pediatric population and 1261 patients (85.9%) were adults. Total incidence rate is slightly higher in adult population than in pediatric population, 5.30 (95% CI, 5.01–5.59) *vs* 4.59 (95% CI, 3.96–5.21) cases/million py. In adult population, the incidence rates of SJS, SJS/TEN, and TEN were 2.84 (95% CI, 2.63–3.06), 0.33 (95% CI, 0.25–0.40), and 2.12 (95% CI, 1.93–2.30) cases per million py, respectively. In pediatric population, the incidence rates of SJS, SJS/TEN, and TEN were 3.59 (95% CI, 3.04–4.14), 0.22 (95% CI, 0.08–0.35), and 0.77 (95% CI, 0.51–1.03) cases per million children py in Spain, respectively. The incidence rates by Autonomous Communities are shown in **Fig 1**, as are the differences between communities (p<0.001).

### Clinical and epidemiological factors

The distribution according to the *ICD-9-CM codes* was: SJS 839 patients (57.2%); TEN 540 patients (36.8%); and SJS/TEN 89 cases (6%). **Table 1**shows the main clinical-epidemiological group of parameters analysed. The sample mean age (±SD) was 52.2 (±26.1) years. Significant differences were observed between age and different diagnoses (p<0.001). The mean age of patients with SJS was 12 years lower than those with TEN. The proportion of women among SJS/TEN and TEN patients was slightly higher (50.6% and 51.3%, respectively), while the number of men with SJS (466, 55.5%) exceeds that of women (p = 0.038). Throughout the study period, the number of patients with the different diagnoses remained stable, (p = 0.460).

**Table 1:** Main clinical-epidemiological data in patients with SJS, SJS/TEN, and TEN.

	SJS, SJS/TEN, TEN n = 1468	SJS n = 839	SJS/TEN n = 89	TEN n = 540	p-value*
**Age, years (Mean ± SD)**	**52.25 ± 26.15**	47.75 ± 27.07	52.35 ± 25.14	59.22 ± 23.22	<0.001*
Pediatric population (<16 years old) (n, %)	**207 (14.1)**	162 (19.3)	10 (11.2)	35 (6.5)	
Adult population (≥16 years old)	**1261 (85.9)**	677 (80.7)	79 (88.8)	5.5 (93.5)	
**Sex (n, %)**					0.038*
Male	**773 (52.7)**	466 (55.5)	44 (49.4)	263 (48.7)	
Female	**695 (47.3)**	373 (44.5)	45 (50.6)	277 (51.3)	
**Diagnostic (n, %)**					<0.001*
Primary/Main diagnosis	**673 (45.8)**	405 (48.3)	61 (68.5)	207 (38.3)	
Secondary diagnosis	**795 (54.2)**	434 (51.7)	28 (31.5)	333 (61.7)	
**Type of admission (n, %)**					0.205
Urgent	**1222 (83.2)**	711 (84.7)	72 (80.9)	439 (81.3)	
Programmed	**246 (16.8)**	128 (15.3)	17 (19.1)	101 (18.7)	
**Readmission (n, %)**					0.103
Re-entry	**1273 (86.7)**	741 (88.3)	74 (83.1)	458 (84.8)	
New episode	**195 (13.3)**	98 (11.7)	15 (16.9)	82 (15.2)	
**Department (n, %)**					<0.001*
Internal Medicine	**481 (32.8)**	310 (36.9)	39 (43.8)	132 (24.4)	
Pediatrics	**172 (11.7)**	145 (17.3)	8 (9.0)	19 (3.5)	
Dermatology	**117 (8.0)**	69 (8.2)	8 (9.0)	40 (7.4)	
Traumatology	**89 (6.1)**	24 (2.9)	3 (3.4)	62 (11.5)	
Plastic Surgery	**82 (5.6)**	7 (0.8)	9 (10.1)	66 (12.2)	
Critical Care Unit	**76 (5.1)**	13 (1.5)	7 (7.9)	56 (10.4)	
Others	**451 (30.7)**	271 (32.3)	15 (16.8)	165 (30.5)	
**Death (n, %)**	**148 (10.1)**	47 (5.6)	11 (12.4)	90 (16.7)	<0.001*
**Hospital stay (mean ± SD)**	**16.38 ± 20.78**	12.90 ± 13.57	15.03 ± 13.23	22.01 ± 28.46	<0.001*
**Hospitalization cost, € (mean ± SD)**	**7885.86 ± 11686.26**	6340.05 ± 7078.85	7492.38 ± 10386.10	10352.46 ± 16319.93	<0.001*

*Statistical significance level of 5% (p <0.05)

Diagnosis of SJS, TEN or SJS/TEN was the primary cause of hospitalization in 673 patients (45.8%), while it was the secondary cause in 795 cases (54.2%), as shown in **Table 1**. The type of admission was urgent in 1222 patients (83.2%), without differences between diagnosis and the types of admission, (p = 0.205). There were 1273 readmission cases (86.7%) and 195 new episodes (13.3%). The mean hospital stay (± SD) was 16.4 (±20.8) days. Statistically significant differences were observed when comparing mean stay and clinical entity: SJS (13 days) *vs* TEN (22 days), (p<0.001).

SJS and TEN in the pediatric population has different epidemiology and outcomes than adults (see **Table 2**). We observed that in pediatric population there is a greater risk or probability/likelihood of SJS, while in the adult population of TEN [OR = 3.45; 95% CI, 2.35–5.06; p<0.001]. We also observe that in the adult population the percentage of men (50.2%) and women (49.8%) is similar, while in the pediatric population the number of boys is double that of girls [ratio, 140/67; OR = 2.1; 95% CI, 1.5–2.8; p<0.001]. The probability of a primary/main diagnosis *vs* secondary diagnoses in pediatric patients is higher than adult patients [OR = 2.7; 95% CI, 1.9–3.7; p<0.001]. Finally, the percentage of deaths among adult patients was higher than in pediatric patients, 11.3% *vs* 2.4% [OR = 5.1; 95% CI, 2.1–12.7; p<0.001].

**Table 2:** Pediatric population (<16 years old) *vs* Adult population (≥16 years old).

		Pediatric population n_1_ = 207	Adult population n_2_ = 1261	p-value*	OR (95%CI)
**ICD-9-CM Code**: (n, %)	695.13	162 (78.3)	677 (53.7)	<0.001*	
	695.14	10 (4.8)	79 (6.3)		
	695.15	35 (16.9)	505 (40.0)		
**Sex:** (n, %)	Male	140 (67.6)	633 (50.2)	<0.001*	2.1 (1.5–2.8)
	Female	67 (32.4)	628 (49.8)		
**Diagnosis**: (n, %)	Primary/Main diagnosis	138 (66.7)	535 (42.4)	<0.001*	2.7 (1.9–3.7)
	Secondary diagnosis	69 (33.3)	726 (57.6)		
**Type of admission:** (n, %)	Urgent	191 (92.3)	1031 (81.8)	<0.001*	2.6 (1.5–4.5)
	Programmed	16 (7.7)	230 (18.2)		
**Hospital stay** (mean±SD)		13.5±15.3	16.8±21.5	<0.032*	5.1 (2.1–12.7)
**Death** (n, %)		5 (2.4)	143 (11.3)	<0.001*	
**Hospitalization cost, €** (mean±SD)		6239.73±5009.09	8156.08±12425.16	0.029*	

*Statistical significance level of 5% (p <0.05)

**Table 3**summarizes the results of the multivariate logistic regression analysis applied to the sample. Variables with most influence in pediatric and adult population were identified: sex [OR = 1.9; 95%CI, 1.3–2.6; p<0.001], clinical entity [OR = 2.8; 95%CI, 1.9–4.2; p<0.001], diagnosis related groups [OR = 2.5; 95%CI, 1.8–3.5; p<0.001] and death [OR = 3.1; 95%CI, 1.2–7.7; p = 0.017]. So, we defined a *pediatric model or pattern* (male with SJS, primary/main diagnosis and low risk of mortality), and an *adult model or pattern* (female with TEN, secondary diagnosis and high risk of mortality).

**Table 3:** Logistic regression model: Pediatric population *vs* Adult population.

Variables in the Equation			B	S.E.	Wald	gf	Sig.	Exp(B)	95% CI for EXP(B)	
	Pediatric population	Adult population							Lower	Upper
Sex	Male	Female	0,631	0,168	14,125	1	0,000	1,879	1,352	2,611
Clinical entity	SJS	TEN	1,048	0,200	27,363	1	0,000	2,851	1,925	4,222
Diagnosis related groups	Primary/Main diagnosis	Secondary diagnosis	0,922	0,165	31,068	1	0,000	2,515	1,818	3,478
Death	Low	High	1,126	0,471	5,720	1	0,017	3,084	1,225	7,762
Constant			0,735	0,123	35,596	1	0,000	2,085		

*Statistical significance level of 5% (p <0.05)

### Mortality cohort

The main mortality data obtained in the cohort are summarized in **Table 4**. Bivariate analysis showed that TEN mortality was 16.7% (90/540), while mortality due to SJS was 5.6% (47/839). Thus, mortality among patients with TEN was three times higher than in patients with SJS [OR = 3.3, 95% CI, 2.3–4.8, p<0.001). Mortality was also higher in regard to a secondary diagnosis [OR = 1.7, 95% CI, 1.2–2.5, p = 0.002). There were no significant differences in mortality either between men (48.6%) and women (51.4%) (p = 0.303) or between urgent and programmed admission (p = 0.178). Finally, as already mentioned above, mortality was five times higher among adult patients than pediatric patients [OR = 5.1; 95% CI, 2.1–12.7; p<0.001].

**Table 4:** Mortality by SJS, SJS/TEN, and TEN.

		Death n = 148	p-value*	OR (95% CI)
**Clinical entity:** (n, %)	TEN	90 (60.8)	<0.001*	3.3 (2.3–4.8)
	SJS	47 (31.8)		
	SJS/TEN	11 (7.4)		
**Population:** (n, %)	Adult	143 (96.6)	<0.001*	5.1 (2.1–12.7)
	Pediatric	5 (3.4)		
**Sex:** (n, %)	Female	76 (51.4)	0.303	1.1 (0.8–1.6)
	Male	72 (48.6)		
**Diagnosis:** (n, %)	Secondary diagnosis	98 (66.2)	0.002*	1.7 (1.2–2.5)
	Primary/Main diagnosis	50 (33.8)		
**Type of admission:** (n, %)	Urgent	129 (87.2)	0.178	1.4 (0.8–2.3)
	Programmed	19 (12.8)		

*Statistical significance level of 5% (p <0.05)

### Hospital costs

**Fig 2:**
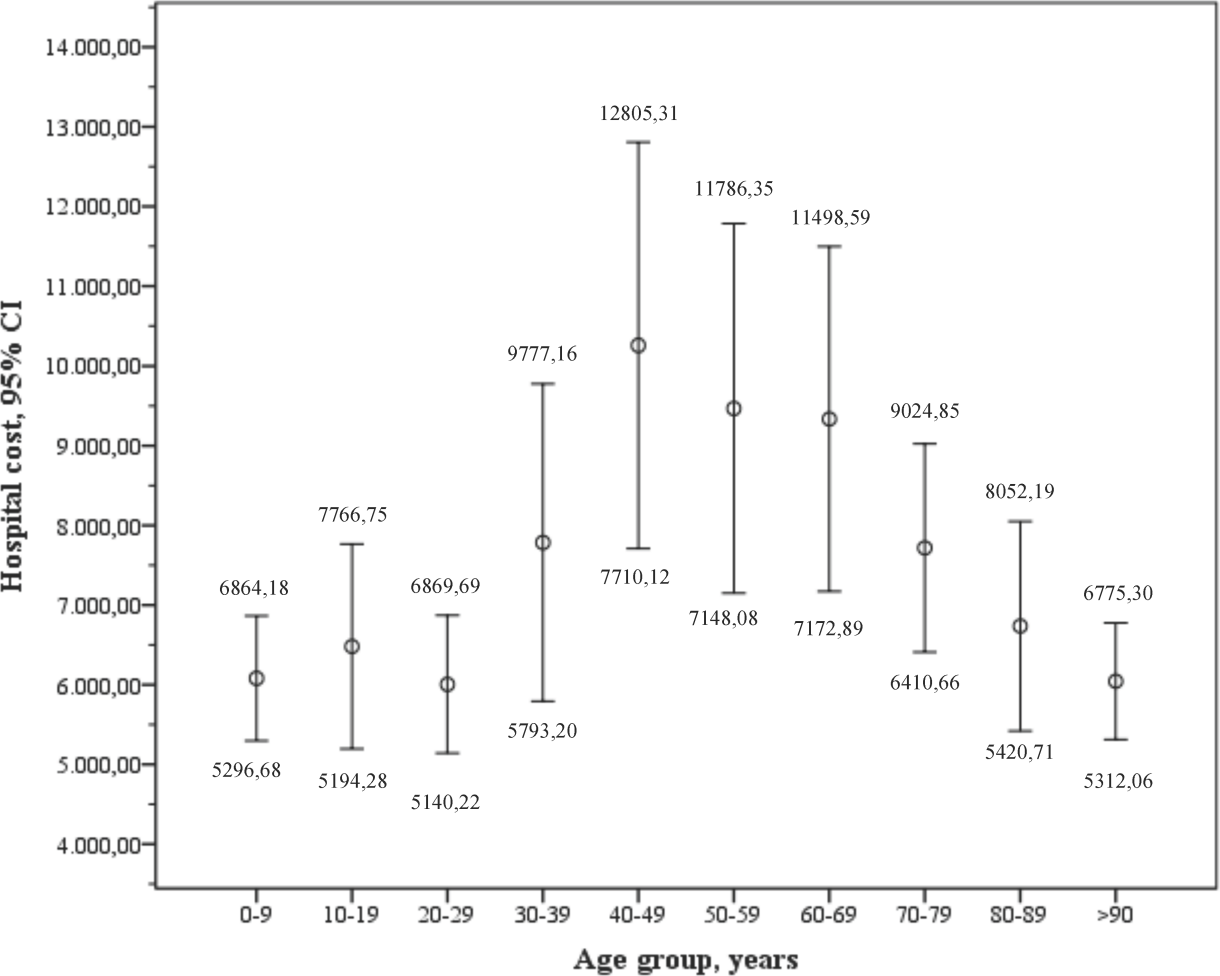
Graph corresponding to *bootstrap* analysis (95 CI for the mean) of the hospital cost data by age.

The total cost of hospital care for patients is estimated at €11.576.456,18 and the average cost per patient (CI 95%) of €7.885,86 (€7.287,56-€8.484,16). The costs for diagnosis and the estimated cost per year included in the cohort are detailed in **Table 5**. There are significant differences (p<0.001) between SJS, SJS/TEN, and TEN (**Fig 2**).

**Table 5:** Costs per year of admission and diagnosis.

	N	Mean	± SD	95% CI for the mean		Total
				Lower	Upper	
**Clinical entity**						
SJS	839	6.340,04	7.078,85	5860,36	6819,73	5.319.301,64
SJS/TEN	89	7.492,38	10.386,10	5304,52	9680,24	666.822,27
TEN	540	10.352,46	16.319,93	8972,89	11732,04	5.590.332,27
**Year**						
2010	237	7.869,84	11.224,73	6433,41	9306,26	1.865.153,14
2011	262	8.824,80	14.732,50	7032,57	10617,02	2.312.098,00
2012	216	8.982,30	14.730,42	7006,75	10957,85	1.940.177,11
2013	282	8.013,14	10.299,75	6805,81	9220,47	2.259.707,52
2014	228	6.517,83	9.049,59	5336,88	7698,78	1.486.067,41
2015	243	7.050,42	8.580,91	5966,10	8134,74	1.713.253,00
**Total**	**1.468**	**7.885,86**	**11.686,26**	**7287,56**	**8484,16**	**11.576.456,18**

## Discussion

The aim of this study is to evaluate the impact of SJS and TEN in the Spanish National Health System during the period from 2010–2015 using CMBD data. Global incidence in Spain in our work was 6.24 x 10^6^ cases per million person-years: 3.65 x10^6^ cases per million person-years for SJS and 2.22 x 10^6^ cases per million person-years for TEN.

At present, it is difficult to assess the *global incidence* of SJS and TEN due to clinical factors: *i)* it is an uncommon pathology; *ii)* cutaneous manifestations may vary according to ethnicity, although there is limited research about ethnic differences in manifestation; *iii)* there is a lack of studies, with different methodologies [1,7,17]; *iv)* many of the studies are old (published over 25 years ago and therefore do not include new drugs), and they usually present the results of a single centre (and not of the general population). Finally, there are structural reasons, such as *iv)* accessibility of the Health Services [18], *v*) prescribing patterns of physicians and *vi*) differences in the marketing of new drugs (which depend on factors such as different administrations).

CMBD is a general registration system that does not require special funds for use, and its codification is under the code of International Code of Diseases ICD-9. The CMBD constitutes a secondary information instrument that provides information regarding the case attended, the hospital activity and its quality.

The NHS provides health care to approximately 95% of the Spanish population (http://www.msssi.gob.es/), so this study provides fairly accurate estimates of the incidence of SJS and TEN in Spain. The incidences remained stable throughout the study period. It is remarkable different incidences between communities in North-Western and South-Eastern Spain. It could be due to socio-economic difference (elderly population in North-Western Spain, higher rent-per-capita in Eastern Spain, etc.) but the design of present work can not clarify this point.

Other studies performed in Germany, France and Korea describe incidences of SJS and TEN of 1.2 ± 6.0 and 0.4 ± 1.2 per million, respectively[7,9–13]. The data described in other European countries, which are similar to Spain in terms of health care, are difficult to compare given the time of the studies and the different methodologies used[7,9–12]. Only the study by *Yang* et al.[13], which showed lower incidences than those described in our study, can be methodologically comparable.

Our data show lower incidence of SJS in pediatric population in Spain than this described in US and similar incidences of SJS/TEN[19]

Our data show a lower TEN mortality rate compared to other studies, whereas the mortality figures of SJS were similar to other estimations[9–13]. Sekula *et al*. described a high mortality after hospital discharge in a follow-up study [14]; due to the methodology of our work, we could not estimate mortality at discharge. In our study, the influence of age on mortality was clear using well-known data, and it was reflected in scales such as SCORTEN[4]. However, it is difficult to attribute the increase in mortality only to age, since there are multiple confounding factors.

The health expenditure varies according to the different economic conditions of each country and the health care systems. In Spain in 2012, the per capita expenditure was €2,668 and the Gross Domestic Product was 8.9% (http:www.oecd.org/spain/), so the total cost of SJS and TEN per person in Spain is significant. Mean cost was higher than total cost of most prevalent in-patient pathology (e.g. pneumonia, heart failure, ischemic stroke) and similar to sepsis cost (http://www.msssi.gob.es/). It should be noted that the estimated cost is only the cost due to hospitalization, and other costs are not accounted for, so the final sum would be higher.

The main limitations of this study are determined by several factors: *i)* the use of sources such as the CMBD for purposes other than research and clinical care; *ii)* the use of the ICD-9 code, which has certain classification limitations with respect to the ICD-10, which is more modern and has fewer qualifying errors[20]; *iii)* in considering only patients in public hospitals and not including non-hospital cases or private centres, for example, those who are ill who are not admitted or who did not receive medical care, in addition to those treated in private hospitals, would be excluded; *(iv)* not being able to access the medical history did not allow us to confirm the diagnosis, identify the possible causal agents involved, assess essential factors such as race, underlying diseases, socioeconomic status, treatment received, or post-discharge assessment; *v)* this study only reflects the patients who died while hospitalized, which could underestimate the mortality; and finally, *vi)* the estimated cost is approximate and less than the real cost, since in this work, only hospital costs have been included.

In conclusion, in this study, older patients have a more severe clinical picture and higher mortality. The overall mortality of both diseases is approximately 10%, with clinical diagnosis and age being the variables with the greatest influence on mortality. Our study describes a similar and stable incidence with respect to other European countries. Additionally, this study shows a high cost for hospitalization due to SJS and TEN. Finally, the CMBD could be a good complementary epidemiological analysis system for the study of hospital management of infrequent and serious diseases such as SJS and TEN.

## Supporting information

S1 Database CMBD SJS & TEN. (SAV)
